# Kallistatin as a Potential Biomarker in Polycystic Ovary Syndrome: A Prospective Cohort Study

**DOI:** 10.3390/diagnostics14141553

**Published:** 2024-07-18

**Authors:** Aslihan Yurtkal, Mujde Canday

**Affiliations:** Faculty of Medicine, Department of Gynecology and Obstetrics, Kafkas University, Kars 36000, Turkey

**Keywords:** diagnostic biomarker, hyperandrogenism, inflammation, kallistatin, oxidative stress, polycystic ovary syndrome

## Abstract

Background: Polycystic Ovary Syndrome (PCOS) is a prevalent endocrine disorder with significant metabolic implications, including an increased risk of cardiovascular diseases and diabetes. Kallistatin, a serine proteinase inhibitor with anti-inflammatory and antioxidative properties, has been identified as a potential biomarker for PCOS due to its role in modulating inflammation and oxidative stress. Methods: This prospective cohort study was conducted at a university hospital’s gynecology clinic. It included 220 women diagnosed with PCOS and 220 healthy controls matched for age and body mass index. Kallistatin levels were quantitatively assessed using enzyme-linked immunosorbent assay (ELISA) techniques. Associations between kallistatin levels and clinical manifestations of PCOS, including hyperandrogenism and metabolic profiles, were examined. Results: Kallistatin levels were significantly lower in patients with PCOS (2.65 ± 1.84 ng/mL) compared to controls (6.12 ± 4.17 ng/mL; *p* < 0.001). A strong negative correlation existed between kallistatin levels and androgen concentrations (r = −0.782, *p* = 0.035). No significant associations were found between kallistatin levels and insulin resistance or lipid profiles. Conclusions: The findings indicate that reduced kallistatin levels are closely associated with PCOS and could serve as a promising biomarker for its diagnosis. The specific correlation with hyperandrogenism suggests that kallistatin could be particularly effective for identifying PCOS subtypes characterized by elevated androgen levels. This study supports the potential of kallistatin in improving diagnostic protocols for PCOS, facilitating earlier and more accurate detection, which is crucial for effective management and treatment.

## 1. Introduction

Polycystic Ovary Syndrome (PCOS) is the most common endocrine-metabolic disorder, affecting 6–20% of the population [1]. The pathogenesis of PCOS is not yet fully understood, with numerous hypotheses suggesting various contributing factors, such as chronic low-grade inflammation and oxidative stress, genetic predispositions, lifestyle influences, post-translational modifications, and ovarian autophagy mechanisms [2,3,4]. Elevated levels of oxidant parameters in individuals with PCOS compared to healthy individuals further suggest a potential role of oxidative stress in the pathophysiology of PCOS [5,6]. Additionally, inflammation caused by high levels of androgens can lead to granulosa cell pyroptosis, resulting in ovarian function decline and tissue fibrosis [7]. The specific causes of PCOS are complex and multi-systemic, such as lack of targeted treatment and different diagnostic criteria and methods, and need to be adjusted according to various clinical symptoms [8]. The abnormal communication of oocytes and follicular cells and the damage of granulosa cells may be the key to PCOS, such as the damage of transregional projection in communication [9]. The prevalence of PCOS is increasing and is linked to a higher risk of metabolic disorders, including cardiovascular disease, atherosclerosis, hypertension, obesity, insulin resistance, metabolic syndrome, Type 2 diabetes, gestational diabetes, hypercholesterolemia, and cancers such as endometrial and possibly ovarian cancer, particularly with hyperinsulinemia [2,10].

PCOS is a complex endocrinopathy with polygenic and polyfactorial origins, exhibiting systemic inflammation and autoimmune characteristics, and is marked by altered steroidogenesis.

Lifestyle errors largely cause it. Inflammation and oxidative stress are crucial in its progression [11].

Kallistatin’s antifibrotic, anti-inflammatory, and antioxidant properties are associated with the etiopathogenesis of PCOS, suggesting a potential role in its complex biological pathways.

Furthermore, emphasizing the significant morbidity of the disease and the potential for diagnostic delays can aid in developing more effective patient management strategies. There is a critical need for biomarkers that effectively address both the reproductive and non-reproductive aspects of this complex syndrome.

Kallistatin in human plasma is recognized as a tissue kallikrein inhibitor and a unique serine protease inhibitor [12,13]. Kallistatin has multiple activities, including vasodilation and inhibiting oxidative stress, inflammation, fibrosis, and apoptosis, primarily by increasing endothelial nitric oxide synthase (eNOS) levels and promoting nitric oxide (NO) formation [14,15]. Through NO formation, kallistatin may protect against vascular injury, senescence, and aging, exhibiting antiangiogenic, antiapoptotic, antifibrotic, antioxidant, and anti-inflammatory effects [13,16]. Notably, kallistatin levels decrease in animal models and humans with inflammatory disorders, suggesting its role in metabolic disorders under excess oxidative stress and inflammation, highlighting its potential as a biomarker for PCOS.

In our research, we aimed to determine kallistatin levels in women diagnosed with PCOS as a diagnostic marker. Our study is unique as the first to explore the relationship between kallistatin levels and the symptoms and signs of PCOS.

## 2. Methods

This study was planned as a prospective cohort study. The ethics committee approval for the study was obtained from the Clinical Research Ethics Committee of Kafkas University with decision number 80576354-050-99/286.

Our study was supported by the Scientific Research Projects Coordination Unit of Kafkas University, with project number 2022-TS-32.

Our prospectively designed study included patients who presented at our hospital’s gynecology outpatient clinic and were diagnosed with PCOS. Key recommendations and updates include diagnosing PCOS using the 2018 International Evidence-based Guideline criteria built on the consensus-based 2003 Rotterdam criteria [17]. Diagnosis requires the presence of two of the following: (i) clinical/biochemical hyperandrogenism, (ii) ovulatory dysfunction, and (iii) polycystic ovaries on ultrasound. PCOM stands for ‘Polycystic Ovary Morphology’, which is a term used in ultrasound diagnostics to describe ovaries that include having a follicle number per ovary of 20 or more follicles on either ovary, observed using high-resolution endovaginal ultrasound technology. An ovarian volume exceeding 10 cm³ may also be used to meet the diagnostic criteria for PCOM [18].

In The 2023 International Guideline for the Assessment and Management of PCOS, alternatively, the anti-Müllerian hormone (AMH) can be used instead of ultrasound [19].

This study’s sample size was determined using G-Power 3.12 software. With a significance level (alpha) of 0.05 and a statistical power of 90%, an effect size of 0.7 was identified, requiring a minimum of 220 subjects per group [20]. All patients’ socio-demographic information was recorded. All clinical examination findings of the patients and the required biochemical data for diagnosis and identification of comorbidities were documented.

The control group comprised 220 individuals who did not fulfill the diagnostic criteria for PCOS and were considered healthy following a comprehensive gynecological evaluation during routine outpatient visits.

The remaining blood samples from routine tests were used to measure kallistatin levels. The control group’s demographic information was recorded. The control and study groups’ socio-demographic characteristics and body mass index (BMI) were closely matched to minimize variability and ensure comparability.

Patients with a family history of systemic diseases (such as cardiovascular disease (CVD), hypertension, diabetes mellitus (DM), or dyslipidemia), inflammatory diseases, or a history of smoking, alcohol, or drug use were excluded from both the study and control groups. Furthermore, patients with ovarian diseases that could affect AMH levels and exhibit a premature ovarian failure profile were also excluded from both groups.

Blood samples were centrifuged, and the obtained sera were stored at −80 °C. All samples were stored at −80 °C. Kallistatin levels were measured using the ELISA method with BT LAB kits, catalog number E3392Hu. Sample, standard, and blank wells were set up. The sample (40 μL) to be tested was added to the sample wells, while the standard sample (50 μL) was added to the standard wells. No reagent was added to the blank well. Anti-SERPINA4 antibody (10 μL) was added to the sample wells, followed by streptavidin-HRP (50 μL) to both the sample and standard wells (not the blank well). The plate was sealed and incubated at 37 °C for 60 min. The liquid was discarded, shaken off, and washed five times. Substrates A (50 μL) and B were added to each well, and then, the plate was sealed and incubated at 37 °C for 10 min. Stop solution (50 μL) was added to each well. A fully automated enzyme-labeled analyzer read each well’s absorbance (OD value) at 450 nm (Allsheng AMR-100 Microplate Reader, Allsheng Instruments Co., Ltd., Hangzhou, China). The intra- and inter-assay coefficients of variation (CV) were calculated to be <8% and <10% for kallistatin. The detection range of the kits was 0.05–200 ng/mL, and the sensitivity was 0.022 ng/mL.

Our study adheres to the Declaration of Helsinki, follows the principles of Good Clinical Practice, and aligns with the ethical guidelines of the respective research subject [21]. Every eligible patient received comprehensive details about our study, and informed consent was obtained. Each of the participating patients acknowledged and signed the informed consent document.

### Statistical Analysis

Demographic information such as race, education level, occupation, income level, past surgical history, and chronic medication use were evaluated using count (n) and percentage (%) values. The Shapiro–Wilk test was used to determine if the continuous variables followed a normal distribution, revealing that none were normally distributed. Therefore, descriptive statistics were expressed as Mean ± SD (standard deviation) and Median (Minimum-Maximum). Mann–Whitney U test was used to compare kallistatin levels between the patient and control groups. Based on ultrasound findings, Kruskal–Wallis non-parametric variance analysis was applied to compare kallistatin values. Spearman’s non-parametric correlation coefficient was used to analyze relationships between kallistatin and variables such as AMH, androgens, Ferriman Gallwey Score (FGS), waist circumference, and BMI. Statistical analyses were performed using IBM SPSS Statistics 21.0 (IBM Corp., IBM, New York, NY, USA) and MS Excel 2007. A *p*-value of <0.05 was considered statistically significant.

## 3. Results

The average age of the individuals participating in the study was 24.01 ± 5.25 years. Of the participants, 6.4% (14) were Caucasian, 73.6% (162) were Turkish, and 20% (44) were from other ethnicities. In terms of education level, 25.9% (57) had primary education, 33.2% (73) had a high school diploma, and 40.9% (90) were university graduates. Occupation-wise, 31.8% (70) were students, 45.9% (101) were housewives, and 22.3% (49) were employed. Regarding income level, 30.9% (68) had a normal income, 64.1% (141) had a low income, and 5% (11) had a high income. In terms of parity, 42.8% (94) were virgins, 29.1% (64) were nulliparous, and 28.1% (62) were multiparous. Systemic diseases were found in 28 individuals (12.7%), and 56 individuals (25.5%) had undergone surgery. A total of 26 individuals (26.4%) were taking chronic medications, with 58 individuals (26.4%) using metformin and 36 individuals (16.4%) using inofolic (Table 1).

The average kallistatin level in patients with PCOS was 2.65 ± 1.84, while the average kallistatin level in the control group was 6.12 ± 4.17. There was a statistically significant difference in kallistatin levels between the patient and control groups (z = 7.377, *p* < 0.001) (Table 2).

No significant relationship was found between kallistatin and AMH (*p* > 0.05). A strong, negative, and statistically significant relationship existed between kallistatin and androgen values (r = −0.782, *p* = 0.035) (Table 3).

There was no statistically significant relationship between kallistatin and insulin, HbA1c, HDL, LDL, total cholesterol, or triglyceride values (*p* > 0.05) (Table 4).

Based on ultrasound findings, no statistically significant difference was found in kallistatin values (χ² = 1.652, *p* = 0.438) (Table 5).

**Figure 1:**
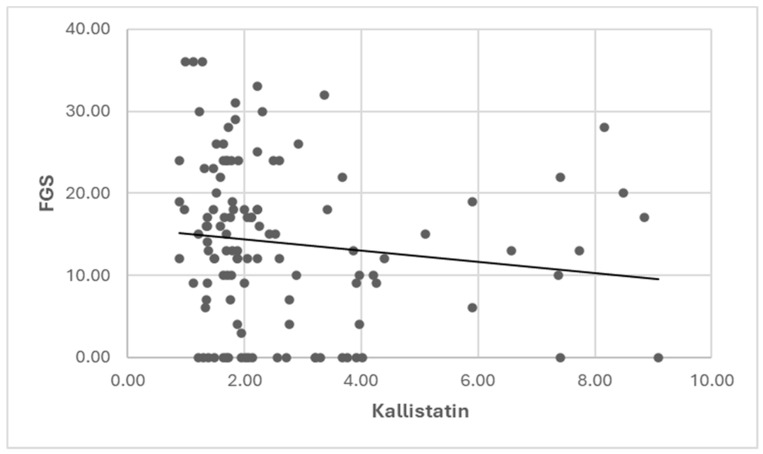
Correlation graph between kallistatin and FGS value in the PCOS group.

A weak, negative, statistically significant relationship existed between kallistatin and FGS values (r = −0.192, *p* = 0.039) (Figure 1). No significant relationship was found between kallistatin and abdominal circumference or BMI values (*p* > 0.05) (Table 6).

No statistically significant difference was found between the kallistatin values of individuals based on clinical parameters (*p* > 0.05) (Table 7).

## 4. Discussion

PCOS is a chronic disorder affecting women during their reproductive years, commonly associated with symptoms such as acne, hirsutism, menstrual irregularities, infertility, obesity, insulin resistance, diabetes, dyslipidemia, hypertension, and metabolic syndrome. These conditions can lead to severe complications, including anovulatory infertility, pathological obesity, autoimmune diseases, inflammation, and long-term risks like endometrial cancer, type II diabetes mellitus, and cardiovascular disease [22]. Additionally, PCOS can cause abnormalities in the female reproductive and immune systems [9].

The pathophysiology of PCOS remains incompletely understood. Inflammation, endothelial damage, oxidative stress, and genetic mechanisms are potential causes of PCOS [5,6]. Due to its complex etiology and wide spectrum of clinical presentations, PCOS necessitates a multifaceted evaluation, extending beyond being just a gynecological pathology, as it has metabolic and psychological effects [23]. Often, each clinical symptom and comorbidity is treated separately, which can delay or obscure a diagnosis of PCOS and limit follow-up care [1,19,24]. Although it can be diagnosed in adolescence, many young individuals may not know they have PCOS or do not receive a diagnosis until they experience infertility [1,25]. The discovery of a diagnostic marker related to PCOS could provide an alternative to unreliable PCOM findings and AMH values, particularly in adolescents. Delays in diagnosis may result in a lack of preventative and comprehensive treatment for both symptoms and comorbidities, further reducing the quality of life and increasing the risk of morbidity. Finding reliable early diagnosis biomarkers is essential to address these challenges effectively [26,27,28,29]. Dissatisfaction primarily arises from delayed diagnosis, lack of specialist referrals, and insufficient information and treatment options. A scoping review of qualitative studies on the diagnostic experience of individuals with PCOS revealed that the prolonged and complex diagnostic process often leaves patients emotionally drained, leading to mistrust in the healthcare system and impacting individual and population health [30]. Additionally, the lack of adequate information at diagnosis can confuse symptoms and the disease, generating feelings of guilt and lack of control [31,32]. These realities underscore the importance of developing reliable diagnostic markers for PCOS.

Kallistatin, an endogenous protein, regulates various signaling pathways and biological functions. In animal models and cultured cells, kallistatin has demonstrated its ability to inhibit inflammation, angiogenesis, oxidative stress, apoptosis, invasion, vascular damage, tumor growth, aging, and metastasis [33].

Our study focused on kallistatin due to its molecular features, which are closely related to the pathogenesis of PCOS. We interpreted our findings to suggest that decreased levels of kallistatin may be associated with an increased risk of inflammation, oxidative stress, and fibrosis. This correlation highlights the potential of kallistatin as a diagnostic marker and its clinical significance in managing PCOS.

The relationship between PCOS and kallistatin as a diagnostic marker holds significant potential, particularly in providing support for diagnostic criteria in patients where these criteria are weak, such as in adolescents where diagnosing PCOS can be challenging. Our study is the first to comprehensively evaluate the clinical manifestations of PCOS in relation to kallistatin. Our research is the only study in the current literature that examines this specific relationship.

In their study, Calan et al. found that circulating kallistatin levels were significantly elevated in women with PCOS compared to controls (6.31 ± 2.09 vs. 4.79 ± 2.26 ng/mL, *p* < 0.001) [34]. Additionally, the researchers observed elevated inflammatory markers hs- C-reactive protein (CRP) and tumor necrosis factor (TNF-α) in women with PCOS. Given the well-established role of inflammation in the etiopathogenesis of PCOS and the recognized anti-inflammatory effects of kallistatin, we chose not to measure inflammation markers in our study to avoid replicating already well-established findings.

Calan et al. also found that kallistatin levels positively correlated with insulin, the insulin resistance index (HOMA-IR), the free androgen index, hs-CRP, TNF-α and carotid intima-media thickness (cIMT) in both PCOS and control groups [34]. This study did not find any difference between the two populations. Our study identified a relationship between kallistatin and androgen levels in PCOS groups, suggesting that kallistatin could be used as a diagnostic marker and for monitoring PCOS, where androgens play a central role in its etiopathogenesis. However, unlike Calan et al., we found no statistically significant correlation between kallistatin levels and insulin-related results in PCOS groups [34].

Furthermore, our study uniquely identified a statistically significant relationship between patients’ FGS scores and kallistatin levels in PCOS groups, indicating that this finding could be promising for monitoring the clinical manifestations of PCOS.

According to the study by Calan et al., multiple linear regression analysis revealed that kallistatin is an independent predictor for cIMT (β = 0.131, 95% CI: 0.114–0.150, *p* = 0.019) [34]. Kallistatin levels may provide useful information regarding cardiovascular risk in women with PCOS [34]. In our study, consistent with the findings of Calan et al. [34], we did not find a statistically significant relationship between kallistatin levels and BMI, insulin, HbA1c, HDL, LDL, total cholesterol, or triglyceride values.

Considering the molecular properties of kallistatin and the results of various kallistatin-related studies in the literature, we formulated our hypothesis for this study by relating kallistatin to the pathogenesis of PCOS. Contrary to the findings of Calan et al. [34], who reported higher kallistatin levels in the PCOS group compared to the control group, we anticipated lower kallistatin levels in the PCOS group. Our study’s results were consistent with our hypothesis.

We can explain the findings of Calan et al. [34] by noting that their patient group had high inflammatory markers, which can be associated with a chronic inflammatory condition like PCOS. Additionally, it is impossible to rule out the influence of acute inflammation, which may have affected their results. Furthermore, the primary hypothesis of Calan et al.’s study was based on cIMT [34], and they ultimately suggested using kallistatin in cardiovascular risk assessment. Given their small PCOS patient sample with high inflammation markers and a focus on cardiovascular risk assessment, it is clear that their study design significantly differs from ours.

Therefore, our study is the first serious research in the literature to evaluate kallistatin as a diagnostic marker.

Obesity is a metabolic condition characterized by chronic inflammation, with elevated levels of pro-inflammatory cytokines, chemokines, and oxidative stress markers [2]. Studies have shown that inflammatory markers and their genetic markers are higher in PCOS patients. Specifically, increased levels of CRP, interleukin 18 (IL-18), TNF-α, interleukin 6 (IL-6), and ferritin have been observed in women with PCOS compared to age- and BMI-matched controls [35]. Given that obesity is a condition characterized by chronic inflammation, a relationship between BMI and kallistatin levels is expected. However, contrary to this expectation, Calan et al. did not find a correlation between BMI and kallistatin levels in their study [34]. Similarly, our study did not detect a significant relationship between BMI and kallistatin levels. No statistical relationship was found between kallistatin levels and the measurements of abdominal circumference or lipid profile data that we evaluated separately.

Based on this information, our study focused on the wide range of symptoms and findings of PCOS, unlike the only study we found in the literature. We aimed to determine whether kallistatin levels in patients could help identify risk factors. We found no statistically significant relationship between kallistatin and menstruation patterns, coronary heart disease, infertility, acne, alopecia, seborrhea, sleep apnea, metabolic syndrome, family history of PCOS, fatty liver, eating disorders, or sleep disorders. In this context, future studies involving larger patient groups and/or groups where PCOS subtypes are distinguished could help clarify these relationships.

The specific causes of PCOS are complex and involve multiple systems. Treatments must be targeted, and different diagnostic criteria and methods exist. These must be adjusted based on the varying clinical symptoms [8]. A literature review has revealed promising results regarding the potential use of kallistatin as both a prognostic biomarker and a therapeutic agent [36,37,38,39,40,41]. Notably, there are significant studies on recombinant kallistatin therapy, particularly its inhibition of signaling pathways in epithelial and endothelial cells, which combats oxidative stress and inflammation [13].

Our study’s primary strength lies in being the first to investigate the correlation between serum kallistatin levels and the wide range of clinics for PCOS. Moreover, our results, consistent with the literature, support using kallistatin as a diagnostic marker.

The primary limitation of our study is our belief that conducting it on larger cohorts could yield more meaningful results. Future studies should be multicentric to increase the study group size and ensure the diversity needed to form PCOS groups, with a particular emphasis on creating groups that separately evaluate adolescents. This would allow our results to be confirmed with comparative patient groups and improve the significance levels of the findings.

## 5. Conclusions

In conclusion, when we consider the results of our study alongside the existing literature, kallistatin is a potential diagnostic and prognostic marker for PCOS. Its biological properties align with the pathophysiological mechanisms of PCOS. Additionally, there are correlations between kallistatin levels and the clinical manifestations of the disease, particularly hyperandrogenism and hirsutism scores.

Future functional research could demonstrate the diagnostic power of kallistatin in all types of PCOS with larger patient numbers while exploring its potential therapeutic role.

## Figures and Tables

**Table 1:** Socio-demographic characteristics of individuals.

		PCOS Group (*n* = 220)
**Age** (year)	Mean ± SD	24.01 ± 5.25
	Median (Min-Max)	22.5 (14–38)
**Race, n (%)**		
Caucasian		14 (6.4)
Turkish		162 (73.6)
Other		44 (20)
**Education Level, n (%)**		
Primary school graduate		57 (25.9)
High school graduate		73 (33.2)
University graduate		90 (40.9)
**Occupation, n (%)**		
Student		70 (31.8)
Housewife		101 (45.9)
Employed		49 (22.3)
**Income Level, n (%)**		
Normal		68 (30.9)
Low		141 (64.1)
High		11 (5)
**Parity, n (%)**		
Virgo		94 (42.8)
Nulliparous		64 (29.1)
Multiparous		62 (28.1)
**Systemic Diseases, n (%)**		
None		192 (74.5)
Yes		28 (12.7)
**History of Surgery, n (%)**		
None		164 (74.5)
Yes		56 (25.5)
**Chronic Medication Use, n (%)**		
None		194 (88.2)
Yes		26 (26.4)
**Metformin, n (%)**		
No		162 (73.6)
Yes		58 (26.4)
**Inofolic, n (%)**		
No		184 (83.6)
Yes		36 (16.4)

**Table 2:** Comparison of kallistatin values according to patient-control grouping.

	PCOS Group (n = 220)		Control Group (n = 220)		Test Statistic	
	Mean ± SD	Median (Min–Max)	Mean ± SD	Median (Min–Max)	z	*p*
**Kallistatin**	2.65 ± 1.84	1.92 (0.88–9.09)	6.12 ± 4.17	4.65 (0.88–15.24)	z = 7.377	**<0.001**

**z**: Mann-Whitney U Test Statistic.

**Table 3:** Relationship between kallistatin and AMH and androgen values in the PCOS group.

	Kallistatin	
	r	*p*
**AMH**	−0.014	0.884
**Androgen**	−0.782	0.035

**r**: Spearman Correlation Coefficient.

**Table 4:** Relationship between kallistatin and insulin, HbA1c, and lipid profiles in the PCOS group.

	Kallistatin	
	r	*p*
**Insulin**	0.046	0.622
**HbA1c**	0.054	0.568
**HDL**	0.110	0.240
**LDL**	−0.076	0.420
**Total Cholesterol**	−0.093	0.319
**Triglyceride**	−0.057	0.543

**r**: Spearman Correlation Coefficient.

**Table 5:** Comparison of kallistatin values according to ultrasound findings in the PCOS group.

	Ultrasound				
	Normal (n = 38)	Bilateral PCOM (n = 66)	Unilateral PCOM (n = 14)	Test Statistic	
	Mean ± SD	Mean ± SD	Mean ± SD	χ^2^	*p*
	Median (Min–Max)	Median (Min–Max)	Median (Min–Max)		
**Kallistatin**	2.95 ± 2.16	2.46 ± 1.66	2.66 ± 1.70	$\chi^{2}=1$.652	0.438
	2.09 (0.88–9.09)	1.79 (0.88–8.84)	2.07 (1.31–7.74)		

**χ²**: Kruskal–Wallis Test Statistic.

**Table 6:** Relationship between kallistatin and FGS, abdominal circumference, and BMI values in the PCOS group.

	Kallistatin	
	r	*p*
**FGS**	−0.192	**0.039**
**Abdominal Circumference**	−0.072	0.440
**BMI**	−0.075	0.426

**r**: Spearman Correlation Coefficient.

**Table 7:** Comparison of kallistatin values according to clinical parameters in the PCOS group.

		Kallistatin		Test Statistic	
		Mean ± SD	Median (Min–Max)	Z *	*p*
**Menstruation Pattern**	Irregular	2.69 ± 1.88	1.94 (0.88–9.09)	z = 0.334	0.739
	Regular	2.53 ± 1.74	1.80 (0.89–8.84)		
					
**Coronary Heart Disease**	No	2.64 ± 1.84	1.92 (0.88–9.09)	z = 0.318	0.765
	Yes	2.95 ± 2.04	2.95 (1.51–4.40)		
					
**Infertility**	No	2.58 ± 1.69	1.88 (0.89–9.09)	z = 0.232	0.817
	Yes	2.80 ± 2.16	1.94 (0.88–8.84)		
					
**Acne**	No	2.61 ± 1.54	1.94 (0.99–8.49)	z = 0.580	0.562
	Yes	2.62 ± 2.02	1.89 (0.88–9.09)		
					
**Alopecia**	No	2.63 ± 1.69	2.00 (0.88–8.16)	z = 0.862	0.389
	Yes	2.60 ± 2.01	1.79 (0.88–9.09)		
					
**Seborrhea**	No	2.66 ± 1.85	1.87 (0.89–8.49)	z = 0.028	0.977
	Yes	2.56 ± 1.80	1.94 (0.88–9.09)		
					
**Sleep Apnea**	No	2.73 ± 1.89	2.00 (0.88–8.84)	z = 0.698	0.485
	Yes	2.44 ± 1.72	1.85 (0.88–9.09)		
					
**Metabolic Syndrome**	No	2.69 ± 1.90	1.94 (0.88–9.09)	z = 0.214	0.830
	Yes	2.26 ± 1.24	1.90 (0.88–5.90)		
					
**Family history of PCOS**	No	2.48 ± 1.60	1.87 (0.89–8.16)	z = 1.152	0.249
	Yes	3.20 ± 2.51	2.21 (0.88–9.09)		
					
**Fatty Liver**	No	2.72 ± 1.90	2.00 (0.89–9.09)	z = 1.094	0.274
	Yes	2.12 ± 1.24	1.79 (0.88–5.90)		
					
**Eating Disorder**	No	2.54 ± 1.66	1.91 (0.88–8.84)	z = 0.093	0.926
	Yes	2.84 ± 2.15	1.95 (0.88–9.09)		
					
**Sleep Disorders**	No	2.71 ± 1.88	2.03 (0.88–9.09)	z = 0.619	0.536
	Yes	2.53 ± 1.77	1.82 (0.88–8.49)		

* Z: Mann–Whitney U Test Statistic.

## Data Availability

The data generated and analyzed during the study are available from the corresponding author. They are not available publicly.
